# Understanding the Lipid and Protein Corona Formation on Different Sized Polymeric Nanoparticles

**DOI:** 10.1038/s41598-020-57943-6

**Published:** 2020-01-24

**Authors:** Tânia Lima, Katja Bernfur, Manuel Vilanova, Tommy Cedervall

**Affiliations:** 1 IBMC-Instituto de Biologia Molecular e Celular, Universidade do Porto, Porto, Portugal; 2 I3S-Instituto de Investigação e Inovação em Saúde, Universidade do Porto, Porto, Portugal; 3 ICBAS- Instituto de Ciências Biomédicas Abel Salazar, Universidade do Porto, Porto, Portugal; 4 Biochemistry and Structural Biology, Lund University, Lund, Sweden; 5 NanoLund, Lund University, Lund, Sweden

**Keywords:** Nanoparticles, Nanomedicine

## Abstract

When in contact with biological fluids, nanoparticles dynamically absorb biomolecules like proteins and lipids onto their surface, forming a “corona”. This biocorona is a dynamic and complex structure that determines how host cells respond to nanoparticles. Despite the common use of mouse models in pre-clinical and toxicological experiments, the impact of corona formed in mouse serum on the biophysical and biological properties of different size NP has not been thoroughly explored. Furthering the knowledge on the corona formed on NP exposed to mouse serum proteins can help in understanding what role it might have in *in vivo* studies at systemic, tissue, and cellular levels. To investigate biocorona formation, different sized polystyrene NP were exposed to mouse serum. Our data show a size- and time-dependent protein and lipid corona formation. Several proteins were identified and apolipoproteins were by far the most common group on the NPs surfaces. Moreover, we observed that cholesterol and triglycerides effectively bind to NP emphasizing that proteins are not the only biomolecules with high-affinity binding to nanomaterial surfaces. These results highlight that further knowledge on NP interactions with mouse serum is necessary regarding the common use of this model to predict the *in vivo* efficiency of NP.

## Introduction

Recent years have witnessed an exceptional progress in research and applications in the field of nanoscience and nanotechnology^1^. This emergent application of nanoparticles (NP) is revolutionizing several aspects of modern medicine due to NP features like longer circulation times, increased solubility, sustained delivery, protection from physical and chemical degradation and also control release of drugs^2–4^. However, a few number of nano-approaches have been accepted for clinical use, while the majority of nanomaterials under clinical research have shown unsatisfactory results^5–8^. In fact, one major obstacle to the successful clinical translation of new nanomedicines is the lack of an accurate understanding of their *in vivo* performance at systemic, tissue, and cellular levels^7^.

Once the nanomaterials interact with biological systems, proteins, lipids and other biomolecules, adsorb on their surfaces promoting the formation of the so-called “biocorona”^9–12^. The formation of the biocorona is a dynamical process, driven by minimization of NP high surface free energy, in which different molecules of the biological fluid compete for the NP available surface^10,13^.

The biocorona is responsible for physical and chemical modifications on the nanomaterial, such as size, aggregation and surface properties, conferring it a biological identity different from the primary synthetic identity^14,15^. Being the NP interface interacting with cells, the biocorona is thought to influence the NP circulation half-time^16^, biodistribution and uptake by cells^17,18^, and host immune response^19,20^, toxicity^21^ and oxidative stress^22,23^.

The adsorption of proteins is influenced specifically by NP properties (size, hydrophobicity, charge and surface chemistry), media (protein source and concentration) and also by the exposure time^10,11,13,24^. Basically, the corona formation is continuous and competitive, through dynamic interactions of proteins and other biomolecules to the nanomaterial surface^25^. The composition changes over time by displacement of earlier adsorbed proteins or by other proteins with stronger binding affinities until equilibrium is reached (“Vroman effect”)^26,27^.

Proteins in the biocorona have been extensively studied. Contrary, the understanding of lipid coronas remains poor. Lipids transportation in blood is mediated by proteins forming complexes called lipoproteins. Lipoproteins are complexes of apolipoproteins, phospholipids, triglycerides, and cholesterol^28^. These molecules are classified by their surface apolipoproteins content and the ratio of the different lipids. Besides lipid transportation, lipoproteins are also associated with other biological processes including coagulation, tissue repair, and immune response^29^. In several studies, proteomic analysis suggested an enrichment of apolipoproteins in the protein corona of nanomaterials. Hellstrand *et al*. reported the interaction of lipids in human plasma with nanomaterials and were the first to describe that lipoprotein complexes as a whole interacted with nanomaterials^12^. Since then only a few other studies have reported lipid, and lipoprotein complexes interacting with a nanomaterial^30–32^. Lipid adsorption was further described in carbon nanotubes after inhalation^33,34^, and it was proposed that lipid adsorption can potentially alter the plasma protein coating pattern^33^. Raesch *et al*. described a synergistic interaction between the prevailing lipids and specific proteins. However, it remains unclear whether the lipoprotein-nanomaterial interactions are mediated by the proteins or the lipid components^35^.

For safe clinical applications, it is accepted that physical-chemical characterizations are needed to comprehend and predict the biological outcome of nanomaterials. The translation between *in vitro* studies to human clinical application usually requires *in vivo* studies, and mouse is the foremost mammalian model for studying human disease and human health. Not surprisingly, mouse models are widely used in nanomedicine research studies, but so far, few studies analyzed the outcome of mouse serum in nanomaterials. Here, the protein corona of carboxylated polystyrene NP (26 nm, 80 nm, 200 nm) was described overtime after BALB/c mouse serum (MS) incubation. Changes in size and zeta-potential were observed as well as the protein composition of biological corona by mass spectrometry. Cholesterol and triglycerides adsorption onto NP surface were quantified revealing differences in lipid corona formation between NP sizes. Our results suggest that studies on mouse serum corona should be carefully considered to improve pre-clinical studies using NP and clinical translation, highlining the need of biocorona formation and composition studies in order to develop safer and efficient nanomaterials for clinical application.

## Results

Over the past years, many studies have been performed describing physicochemical and biological features of the protein corona on NP. However, although murine strains are the most widely used models in biomedical research, the knowledge about the protein corona formed on NP exposed to mouse serum is still scarce. Thus, studies about how protein corona influences NP functionality and toxicity in different research models are urgent. In biomedical studies, NP are usually exposed to different protein sources. *In vitro* tests are usually performed in culture media supplemented with fetal bovine serum while in *in vivo* studies NP are exposed to animal model’s serum (usually mouse or rat) and, when in clinical studies, to human serum. However, so far, less than thirty studies about mouse serum corona have been published, and, to our knowledge, none using polystyrene despite its common use as a model nanoparticle. Contrastingly, hundreds of corona studies were performed using human serum corona. The mouse is the research model mostly used worldwide. Accurate and more extensive information concerning this model would be useful to understand in greater detail, preclinical studies. This would help determine what responses observed using *in vitro* and *in vivo* could be translated to clinical trials, and in that way improving the outcomes of nanomedicine projects.

Polystyrene NP are commonly used due to their commercial availability, high quality and a wide variety of size and surface chemistries^36,37^. These polymeric NP have been reported to enter different cell types. The specific uptake pathways, as well as the uptake rates, have been shown to be cell type-, NP size-, and shape-dependent, and are also related to the surface chemistry of the particles and their hydrophobicity^38^. Here, polystyrene NP sized 26, 80 and 200 nm were used as experimental model.

A comprehensive characterization of the NP was performed using transmission electron microscopy (TEM) in order to measure particles in a dry state, and dynamic light scattering (DLS) and differential centrifugal sedimentation (DCS) to obtain hydrodynamics diameter in PBS (Table 1). The TEM analysis revealed that NP have a diameter in accordance with the nominal information given by the manufacturer. The size was also confirmed through DLS and DCS in buffer. The smaller COOH-PS NP (26 nm) showed a higher polydispersity index (PDI) (0,361 ± 0,009) and the 200 nm a lower PDI (0,015 ± 0,010).

**Table 1:** Polystyrene nanoparticle characterization.

Manufacturer nominal size (nm)	Particle Diameter (nm)			PDI	ζ-potential ± SD (mV)	Surface área (μm^2^g^−1^)
	TEM	DLS	DCS			
200	199,5 ± 10,5	183,0 ± 1,8	214,8 ± 3,0	0,015 ± 0,010	−35,2 ± 2,0	2,9 × 10^13^
80	78,1 ± 4,02	76,4 ± 0,8	89,1 ± 0,1	0,022 ± 0,022	−35,8 ± 1,4	7,1 × 10^13^
26	24,2 ± 3,20	26,4 ± 0,4	30,7 ± 7,4	0,361 ± 0,009	−27,3 ± 1,4	2,2 × 10^14^

The average size of the different NP was determined in the dry state (TEM) and an aqueous solution (DLS and DCS) with a COOH-PS concentration of 0,5 mg ml^−1^. PDI values were determined by DLS. Values represent mean ± SD from three independent experiments.

**Figure 1:**
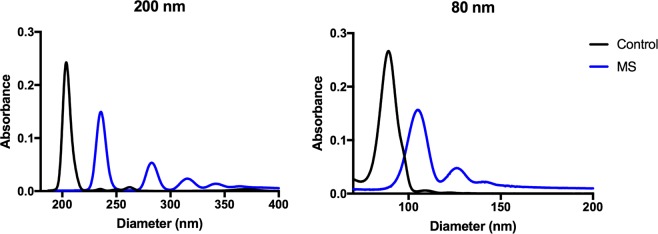
Size distribution of COOH-PS nanoparticles in PBS (black) and MS (blue). Mean by DCS. Measurements were performed by DCS in PBS and 1 h after MS incubation.

Next, we incubated the NP in MS. We observed by DCS (Fig. 1) that in the presence of MS COOH-PS nanoparticles tend to aggregate over time. The observed increase in NP size determined by DLS (Supplementary Fig. 2) and the concurrent increase in the polydispersity seem to be closely related with protein corona formation after incubation with serum. The aggregation process was more pronounced for 80 nm and 26 nm COOH-PS (Supplementary Figs. 2 and 3). NP analyzed by DCS are increased in size and, additional larger populations are seen after incubation in MS (Fig. 1). However, mixed populations in the samples could explain the increase in polydispersity observed by DLS.

The sedimentation rate is a result of size, density and shape effects, which makes it difficult to interpret the cause for changes in the sedimentation rate. For example, when working with polystyrene, adsorbed proteins will increase the size and increase the average density of the formed complex. The two changes will have the same effect on the sedimentation rate. Adsorbed lipids and apolipoprotein particles will also increase the size but the average density of the formed complex will decrease. In this case the changes will have opposite effects on the sedimentation rate. Despite long sedimentation time it was possible to analyze the 26 nm nanoparticles by DCS (Supplementary Fig. 4). However, it was not possible to analyze the 26 nm nanoparticles after MS incubation, probably due to the presence of free proteins and lipoprotein particles with sedimentation speeds similar to the nanoparticles. The size-dependent aggregation observed could be associated with size-related properties of NP, such as surface area and surface reactivity. He *et al*. described that the effect of ionic strength on NP aggregation increases with the decreasing of NP size and that the same ionic strength has a higher impact on aggregation behavior of smaller size NP^39^. In fact, large specific surface areas present high reactivity and a large fraction of surface atoms which were shown to actively affect nanoparticle stability^39^.

As stated before, the absorbed protein corona on the surface of NP change their surface properties, such as zeta-potential and hydrophobicity altering the nanomaterials behavior. Therefore, the zeta-potential of COOH-PS was evaluated before and after MS incubation. Zeta-potential values showed that all the samples were negatively charged. Zeta-potential of COOH-PS significantly changed after MS incubation (Supplementary Fig. 5). The naked COOH-PS NPs showed a negative surface charge between −27,3 and −35,8 mV, whereas after 1 h incubation with MS (but prior to washing) zeta-potential increased to −10,5 mV (200 nm), −7,3 mV (80 nm) and −9,7 mV (26 nm). After washing and re-dispersion of the COOH-PS pellets, the zeta-potential of all COOH-PS showed a slight decrease. This can be due to loosely bound proteins being washed away, changes in the dispersion, or to a fraction of the protein covered COOH-PS being not pelleted during centrifugation. These results are in accordance with Tenzer *et al*., that showed that proteins displaying a negative charge overall at physiological pH constituted the majority of the corona components for all NP. Therefore, accumulation of proteins on the COOH-PS NP surface leads to changes in the surface charge depending on bound protein amount^13,40^. This is not surprising as the majority of proteins present in MS are negatively charged^13^.

**Figure 2:**
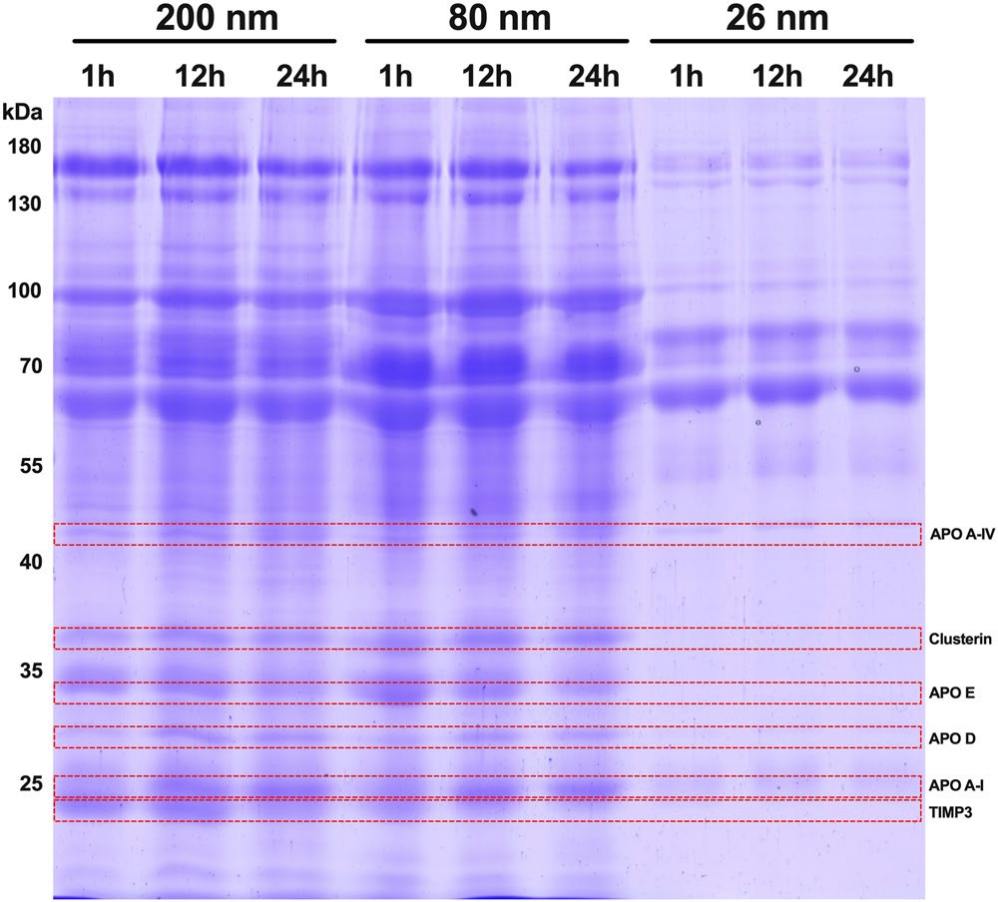
Size dependence differences in protein corona of COOH-PS NPs after MS incubation. NPs (200 nm, 80 nm, 26 nm) were incubated at 0,5 mg ml^−1^ (mass normalized samples) with MS during 1 h, 12 h and 24 h. NP were separated from free proteins by centrifugation and proteins adsorbed to COOH-PS NP was resolved by 10% SDS-Page gel and stained with Coomassie. Full length gel is presented in Supplementary Fig. 6.

Next, we investigated what proteins bound to COOH-PS NP. After 1 h, 12 h, and 24 h of incubation with MS, the protein/particles complexes were collected by centrifugation, the pellet washed and the protein separated by gel electrophoresis. Analysis of Coomassie-stained gels showed a broad variety of proteins in the corona on all COOH-PS NP (Fig. 2). The proteins were identified by mass spectrometry, (Supplementary Fig. 6 and Supplementary Table 1). Proteins further discussed are indicated in Fig. 2. In the control experiment, in which MS was incubated without NP but treated in exactly the same way, no proteins were detectable.

The analysis of the protein patterns reveals several interesting results (Fig. 2). Firstly, there is a size-dependent difference. The individual protein patterns of each sized NP are similar but not indistinguishable. In fact, the 80 nm COOH-PS NP present a more intense protein profile compared with the other two sized samples. The smallest, 26 nm COOH-PS NP appear to bind fewer proteins. Notably, the apolipoproteins are much less abundant and one, apolipoprotein E (APO E), cannot be detected in 26 nm NPs. It cannot be ruled out that a subpopulation of the 26 nm particles, due to changes in density after possible binding of lipoprotein particles, do not pellet. However, the presence of albumin and indicate that the 26 nm nanoparticles are pelleted. Secondly, there are time-dependent differences at the same MS concentration for single COOH-PS NP. The identified band related to protein APO E on 200 and 80 nm COOH-PS NP, showed a time-dependence profile becoming tenuous after 24 h incubation. The same time-dependent pattern was observed for Metalloproteinase inhibitor 3 (TIMP3) in 200 nm and 80 nm COOH-PS NP. In opposite, APO A-I that was barely detected after 1 h incubation, increased in intensity over time. Similarly, clusterin, reported having a preventive role in the uptake of sterically protected NP by macrophages^41^, also increased overtime on 80 nm COOH-PS NP.

**Figure 3:**
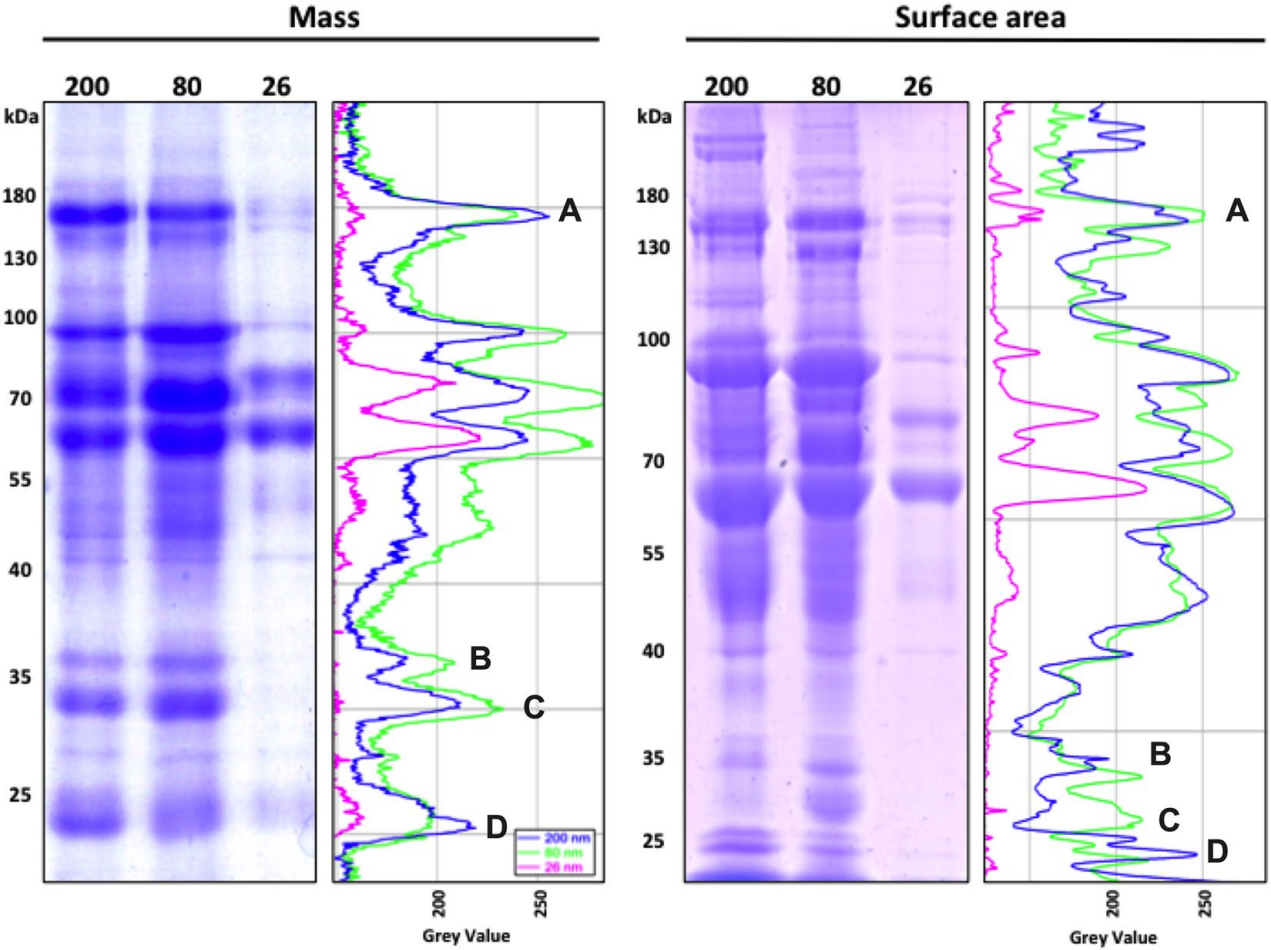
Size-dependent differences around COOH-PS NP by SDS-page gel. 200 nm, 80 nm and 26 nm COOH-PS NPs were incubated with MS at 0,5 mg ml^−1^ or for 1 h at 37 °C. The protein corona was resolved by 10% acrylamide SDS-page gel and a densitometry analysis using Image J/FIJI. A- Myosin-9, B- Clusterin, C- APO E, D- APO A-I. Full lenght gels are presented in Supplementary Figs. 7 and 8.

In the above experiment, the proteins binding after 1 h incubation in MS was compared with the same surface area or total mass constant between the three-sized COOH-PS NP. Densitometry analysis (Fig. 3) showed differences between the COOH-PS NP when analyzed at the same time (1 h) serum incubation. This analysis showed that 80 nm COOH-PS NP adsorbed most of the different proteins, except for two. The 200 nm COOH-PS bind more of protein Myosin-9 and APO A-I. The 26 nm COOH-PS NP, as already described above, had fewer proteins absorbed on their surface. Additionally, after 24 h, the decrease in adsorbed APO E and the increase in APO A-1 were similar to what is described above (Supplementary Fig. 9).

**Figure 4:**
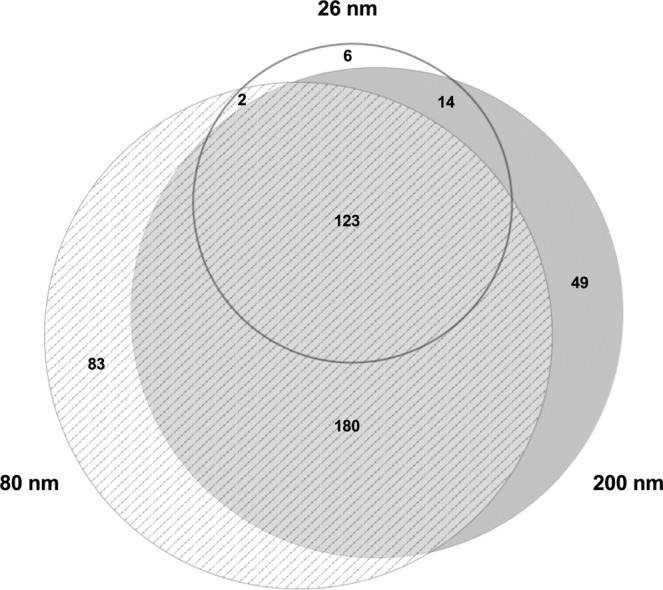
Venn diagram representing the distribution of proteins found to associate with COOH-PS NP following incubation with MS.

A comparison of the 20 most abundant proteins found on COOH-PS NP corona reveals some similarities between the three studied NP sizes (Supplementary Table 1). A proteomic analysis showed that 49 proteins bind uniquely to 200 nm COOH-PS NP, whereas 83 were found to only bind 80 nm COOH-PS NP and there are 6 unique proteins binding to 26 nm COOH-PS NP (Fig. 4). These differences observed through mass spectroscopy analysis are in accordance with the results already discussed related with protein profiles identified through SDS-page.

These differences in protein profiles between different studied COOH-PS NP provide a singular protein corona signature for each of the different sized NP. The biological importance for some of the proteins in the corona is described in the Supplementary Box 1.

**Figure 5:**
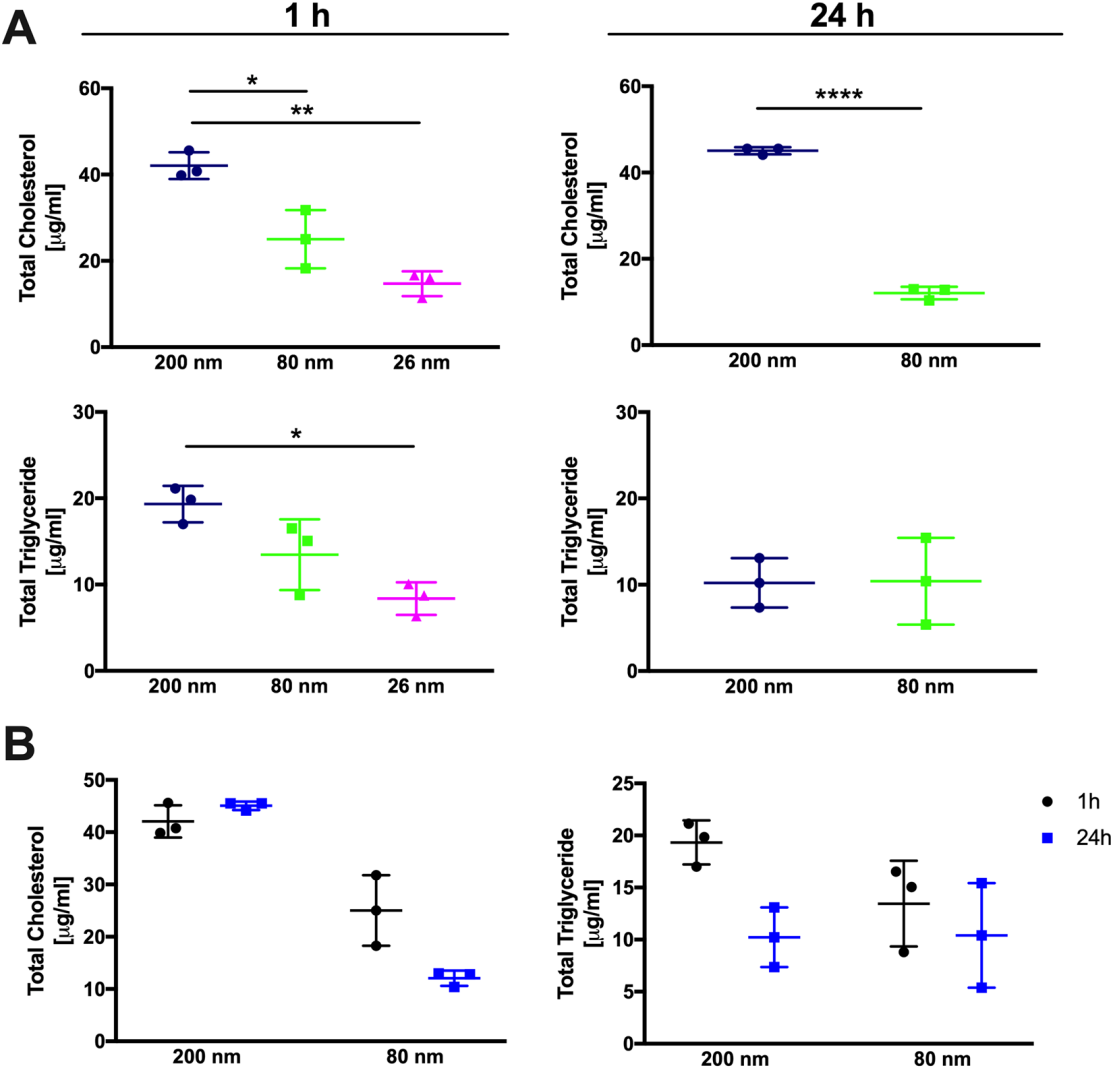
Lipid quantification on COOH-PS NP after MS incubation. Levels of triglycerides and cholesterol in the corona were quantified after 1 h and 24 h incubation with MS in COOH-PS NPs normalized to 5 × 10^10^ μm^2^ surface area. (**A**) Overtime comparison between 200 nm and 80 nm lipid corona. (**B**) Each condition was set in triplicate. Bars correspond to means plus SD. One-way ANOVA with Bonferroni post Hoc test. (*P < 0,05; **P < 0,01; ****P < 0,0001).

Considering the abundance of apolipoproteins and also the size-dependent differences in the coronas composition we found it interesting to quantify the amount of triglycerides and cholesterol in the corona (Fig. 5). To ensure that there are no differences in the experimental routine, the pellets were split into two equal parts before cholesterol and triglyceride quantification. COOH-PS NP were incubated in MS and bound lipids were separated from free lipids by sucrose density centrifugation as described in material and methods. The measurements were performed using the same total surface area or total mass.

The amount of cholesterol binding is independent of surface area but related to NP size. The 200 nm NP had the highest content of cholesterol independent on its surface area or total mass that was kept constant (Fig. 5 and Supplementary Fig. 10). This pattern was still observed after 1 h and 24 h of MS incubation indicating that one additional factor that may influence the cholesterol binding is the particle size and consequent surface curvature^12^. Nevertheless, a slight decrease of cholesterol was observed after 24 h, indicating a possible dissociation of these lipids.

In contrast, the levels of triglycerides suggest a relationship with the NP surface area. When the surface area is normalized, 200 nm COOH-PS NP showed a higher content of triglycerides. However, when analyzed based on NP total mass, no significant differences were observed between 200 nm and 80 nm samples. The triglycerides level decreased between 1 h and 24 h (Supplementary Fig. 10). Interestingly, the same was observed for APO E levels. APO E is associated with VLDL lipoproteins, which constitute approximately 50% triglycerides and 22% cholesterol. It can be speculated that the decrease in APO E levels explains the reduction in triglycerides.

The molar ratio (Table 2) of cholesterol and triglycerides bound to each NP sample was calculated based on data from Fig. 5. The molar ratio varied between 1,15 to 4,42. This is similar to what previously reported on copolymer particles and a ratio indicative for HDL^12^. The marked differences between 200 nm and 80 nm indicated that specific lipoprotein particles bound to different COOH-PS NP.

**Table 2:** Molar ratio of lipids (cholesterol/triglycerides) on COOH-PS NP after 1 h and 24 h MS incubation.

		200 nm	80 nm	26 nm
Surface area	1 h	2,18	1,86	1,75
	24 h	4,42	1,16	—

Experiments were performed at 37 °C with the same total surface area (5 × 10^10^ μm^2^).

It remains an unsolved issue whether the lipoprotein-NP interaction is mediated by proteins, in particular, apolipoproteins, or by the lipid components. It has been suggested that binding of lipoprotein particles, in particular, HDLs, may be mediated by APO A-I, due to the fact that it is the major component of HDL complexes. Moreover, it was proposed that copolymer NP bind complete HDL complexes, and may be recognized by living systems as HDL complexes^12^.

We observed that abundance of APO A-I increases over time and the total cholesterol also increases in 200 nm NP. On the other hand, APO E decreases after 24 h of incubation similarly as triglycerides levels. In fact, these results suggest that 200 nm sized NP could adsorb intact lipoproteins complexes after MS incubation. However, the same does not happen with 80 nm NP, and no relation between apolipoproteins and cholesterol or triglycerides was observed. This observation agrees with those recently reported by Muller *et al*., that after contact with polymeric NP, a disintegration of lipoproteins adsorption of lipids occurs^42^.

We also cannot exclude whether washing procedures can influence the lipid quantification analysis promoting the disintegration or elimination of lipoprotein complexes. Moreover, the release of APO E and the increasing levels of APO A-I on COOH-PS NP corona could contribute to rearrangement of protein and lipid corona.

So far, related with lipoprotein and NPs interactions, two situations should be considered in terms of lipoprotein adsorption: (i) intact adsorption of lipoprotein complexes or (ii) disintegration of complexes and consequent adsorption of individual structures.

Overall, we observed that proteins are by far not the only biological molecules that dynamically interact with COOH-PS NP and it is essential to consider also lipoproteins. Besides, to an efficacious use of nanomaterials in biomedical applications, the interaction of lipoproteins should be cautiously pondered, considering the ability of lipoproteins and its constituent apolipoproteins to aid uptake into target cells affecting biodistribution, clearance, and the consequent biological fate.

## Conclusions

The proteins corona has been demonstrated to have an enormous impact on the biological behavior of NP. However, this study shows that proteins are by far not the only type of biological molecules interacting with nanomaterials. So far, little is known about the corona lipid content and also the biological impact of lipids onto NP surface.

We demonstrate with this work, that in a physiological environment (mouse serum), a complex protein corona formed rapidly, changed with time and was NP size dependent. The formation of protein corona, affected the physical and chemical NP characteristics, increasing hydrodynamics diameter and modifying NP surface charge. The adsorbed protein corona has different protein patterns depending on NP size. Interestingly, 80 nm COOH-PS NP bind the highest amount of proteins. However, all protein coronas contained different patterns of proteins related with immune response, lipid metabolism and transport capacity to modulate in distinct ways NP-cell interactions involved in their uptake and fate into cells and tissues.

In addition to proteins NP bind lipids with high specificity. Lipids are natural blood constituents, mostly transported in lipoprotein particles, which emphasizes the relationship between protein and lipid corona analysis. The pattern of apolipoproteins, cholesterol and triglycerides supports the conclusion that HDL rather than LDL binds to polystyrene under physiological conditions. We also observed that lipid corona is extremely dynamic overtime compared to the protein corona (Fig. 5). The need to study the lipid influence on nanomaterials is therefore urgent. Apolipoprotein complexes are very important for the metabolism and increased knowledge of their interaction with nanomaterials should enable better use of nanomaterials in clinical applications.

These results suggest that mouse serum protein corona studies are extremely pertinent and indispensable to understand *in vivo* preclinical studies using nanomaterials.

The concept of the personalized biomolecular corona has arisen suggesting that NP coronas should be characterized individually to specific conditions. These studies can help predict adverse effects according to protein and lipid functions and give us information about the effect on the nanoparticles-biological interface, which may have important implications in nanomedicine and nanotoxicology.

## Methods

### NP and mouse serum

Carboxyl (-COOH) surface modified PS NPs were purchased from Bang laboratories, (USA). All the PS nanoparticles were extensively dialyzed against distilled water before use. BALB/c MS)was purchased from Innovative Research (USA) and sterile filtered through a 0,22 μm filter before use.

### Characterization of polystyrene NP

COOH-PS NP were diluted with either water, phosphate buffer saline (PBS), and MS. The analyses were conducted between 0 h to 24 h after incubation with MS. The hydrodynamic size of NP was measured by DLS on DynaPro Plate Reader II (Wyatt instruments, USA), at 37 °C, with 10 acquisitions per sample. Before the analysis of MS samples, the refractive index and viscosity were adjusted to 1,333 and 1,330 cP respectively.

DCS was performed using DC24000 disc centrifuge (CPS Instruments, USA), with a linear 8–24% sucrose gradient at 24000 RPM. Data were analyzed for sizes between 1 μm to 20 nm assuming spherical particles with a density of 1,06 g cm^−3^.

For negative staining TEM, samples were mounted on Formvar/carbon film-coated mesh nickel grids (Electron Microscopy Sciences, Hatfield, PA, USA). 1% uranyl acetate was added on to the grids and visualization was carried out on a JEOL JEM 1400 TEM at 120 kV (Tokyo, Japan). CCD digital camera Orious 1100 W Tokyo, Japan,was used to digitally record images.

To analyze the effect of MS on nanoparticle zeta-potential, nanoparticles suspensions were incubated with MS for 1 h. After incubation, the samples were pellet (20 min, 30000 × g, 20 °C), resuspended in PBS and analyzed by Zetasizer. Furthermore, to evaluate the effect of washing steps in surface charge, the pellets were washed three times with PBS under the same conditions as the first centrifugation. After the first centrifugation, all NP samples were transferred from the incubation tube into a new test tube to avoid false positives signals caused by proteins adsorbed to the wall of the incubation tube. The zeta-potential and polydispersity index of the particles was determined by DLS at 25 °C with a Malvern ZetaSizer Nano ZS particle analyzer. The Malvern Dispersion Technology Software (DTS) was used with monomodal mode data processing was used to determine average zeta-potential (mV) and error values. All measurements were set in triplicate.

### Size selective separation of NP bound proteins

NP suspensions were incubated with MS for 1 h, 12 h, and 24 h. The samples were centrifuged (20 min, 30000 × g, 20 °C) to pellet the particle–protein complexes. The pellet was washed in PBS, transferred to a new vial, and centrifuged again to pellet the particle–protein complexes. This procedure was repeated three times. After the third washing step, the proteins were eluted from the particles by adding SDS-sample buffer (dH_2_O, 0,5 M Tris-HCl, Glycerol, 10% SDS, 2-mercaptoethanol, 1% bromophenol blue). Corona proteins were separated by electrophoresis through 10% acrylamide SDS/PAGE 1D gels. Each gel run included a molecular weight ladder standard, PageRuler Prestained Protein Ladder #26616 (Thermo Scientific). Thereafter, the PAGE gel was stained with Coomassie during 2 h.

### Protein identification by mass spectrometry

Protein digestion were performed according with Shevchenko *et al*.^43^. 17 gel segments from the SDS gel were cut into pieces (1 × 1 mm) according to the scheme in Supplementary Fig. 1.

Peptide separation and mass spectrometry were performed according with Gunnarsson *et al*.^30^ Raw files containing 7000 scans each were converted to mgf-format by Mascot Distiller (version 2.6) and identification of proteins was carried out with the Mascot Daemon software (version 2.5). The files were searched against Mouse in the database Swiss Prot.

### Determination of corona total triglyceride and cholesterol concentration

For a serum-free analysis of NP corona of COOH-PS NPs after MS incubation, the samples were centrifuged in 20% sucrose solution. After incubated in MS the mixture was loaded on top 1 ml of 20% sucrose solution and the tubes centrifuged during 30 min at 30000 g, 4 °C. After centrifugation, the supernatants (serum and sucrose solution) were discarded and the pellet (COOH-PS NP) washed twice with PBS to remove excess of sucrose.

Total triglyceride concentration analysis was performed by mixing Triglyceride Reagent, a lipase reagent to cleave triglycerides to glycerol, with Free Glycerol Reagent, a chromogenic enzyme-based solution, according to manufacturer instructions (Sigma-Aldrich, USA). The COOH-PS were pelleted and the absorbance of supernatants measured at 540 nm using ProbeDrum (Probation Labs, Sweden).

The amount of cholesterol present onto NP surface after 1 h and 24 h of MS incubation was determined using the Amplex Red Cholesterol Assay Kit (Invitrogen, UK) according to the manufacturer’s instructions (detection limit: 80 ng/ml).

### Statistical analysis

The data analysis was performed using GraphPad Prism 8 (GraphPad Software Ins.). The data were presented as mean ± standard deviation of at least 3 independent experiments.

## Supplementary information

Supplementary data.

## Data Availability

The datasets generated during and/or analyzed during the current study are available from the corresponding author on reasonable request.
