# Supplementary material for: Understanding the Lipid and Protein Corona Formation on Different Sized Polymeric Nanoparticles
Source: Sci Rep. 2020 Jan 24;10:1129. doi: 10.1038/s41598-020-57943-6 (PMC6981174; doi:10.1038/s41598-020-57943-6)
Supplement: Supplementary file 1 — Supplementary data. [file 41598_2020_57943_MOESM1_ESM.pdf]

Understanding the lipid and protein corona formation on different sized polymeric nanoparticles

Tânia Lima*[1,2,3], Katja Bernfur[4], Manuel Vilanova[1,2,3] and Tommy Cedervall[4,5]

1- IBMC-Instituto de Biologia Molecular e Celular, Universidade do Porto, Porto, Portugal.

2- I3S-Instituto de Investigação e Inovação em Saúde, Universidade do Porto.

3- ICBAS-Instituto de Ciências Biomédicas de Abel Salazar, Universidade do Porto, Porto, Portugal.

4- Biochemistry and Structural Biology, Lund University, Lund, Sweden.

5- NanoLund, Lund University, Lund, Sweden.

Supplementary material

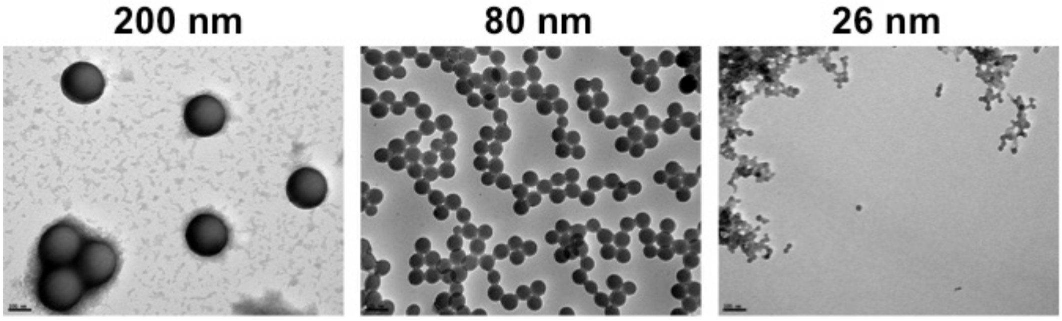

Supplementary Figure 1: COOH-PS nanoparticles structure analysis. Representative micrographs assessed by Transmission Electron Microscopy (TEM).

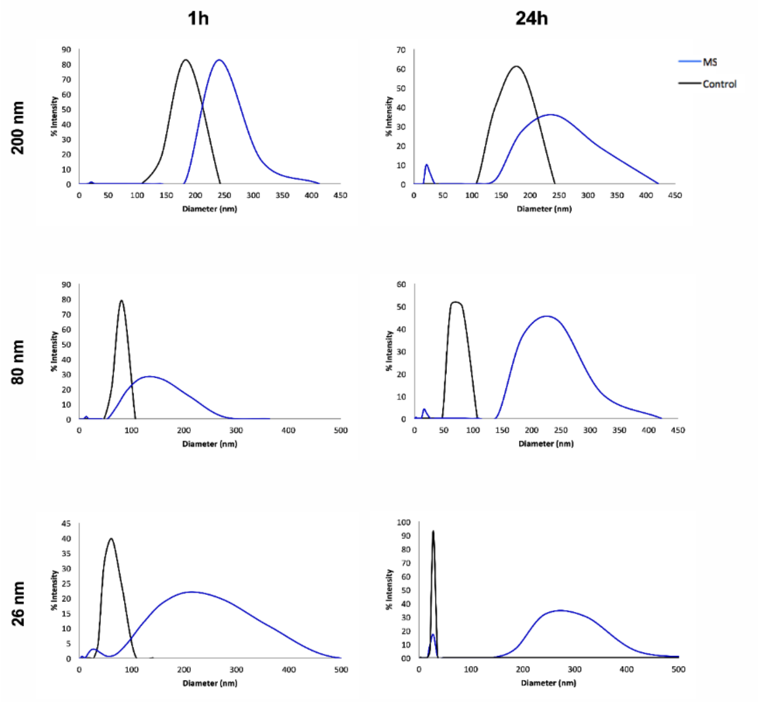

Supplementary Figure 2: Hydrodynamics radius of 200 nm, 80 nm, and 26 nm COOH-PS NP in PBS (control) and MS after 1h and 24 h incubation. Measurements by DLS were conducted automatically every 90 min, during 24h at 37°C. All data is shown in Supplementary Figure 2. The analysis was performed adjusting the refractive index and viscosity to 1,333 and 1,330 cP respectively, to MS samples.

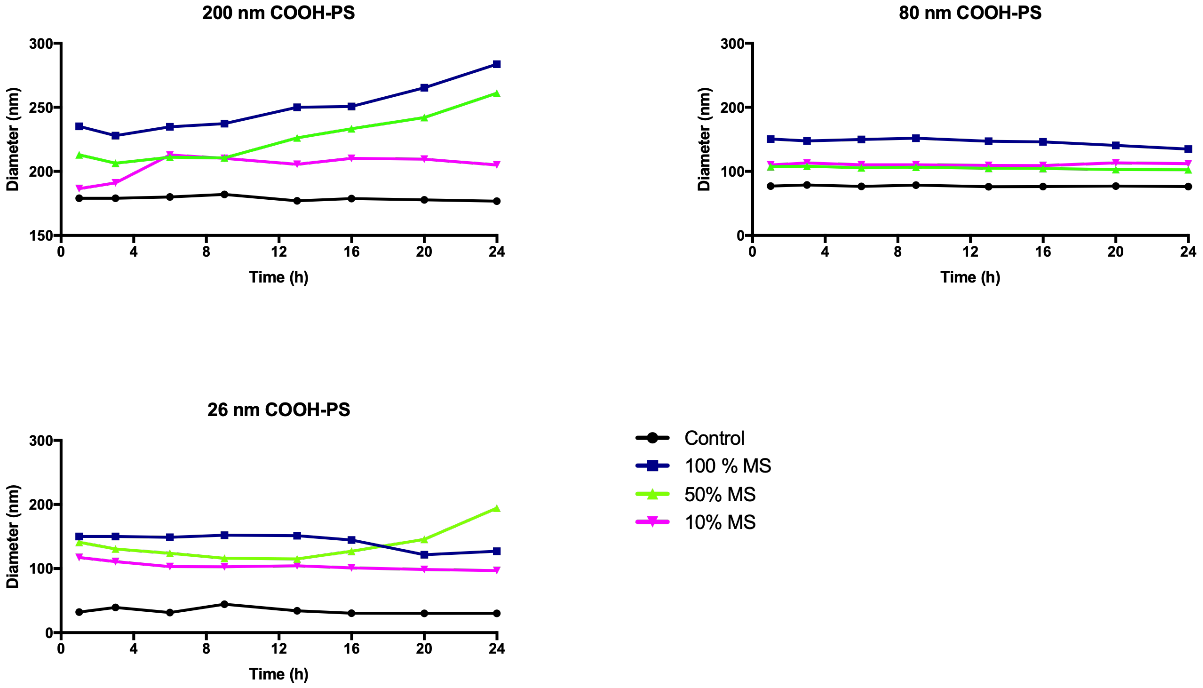

Supplementary Figure 3: Hydrodynamics radius of 200 nm, 80 nm and 26 nm COOH-PS NP in PBS (control) and MS. Measurements by DLS were conducted automatically every 90 min, during 24h at 37°C. The analysis was made adjusting the refractive index and viscosity to 1,333 and 1,330 cP respectively, as described to MS.

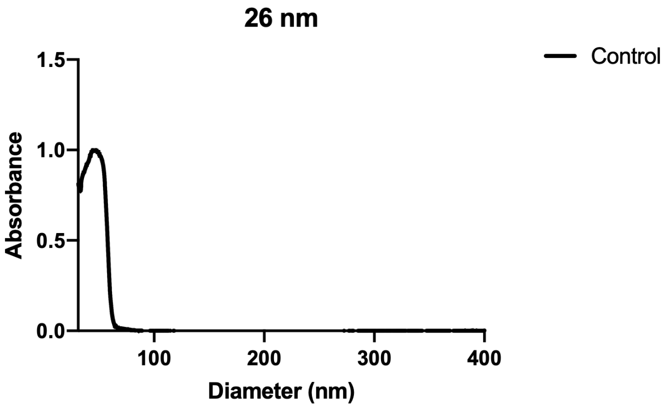

Supplementary Figure 4: Size distribution of COOH-PS 26 nm nanoparticles in PBS by DSC.

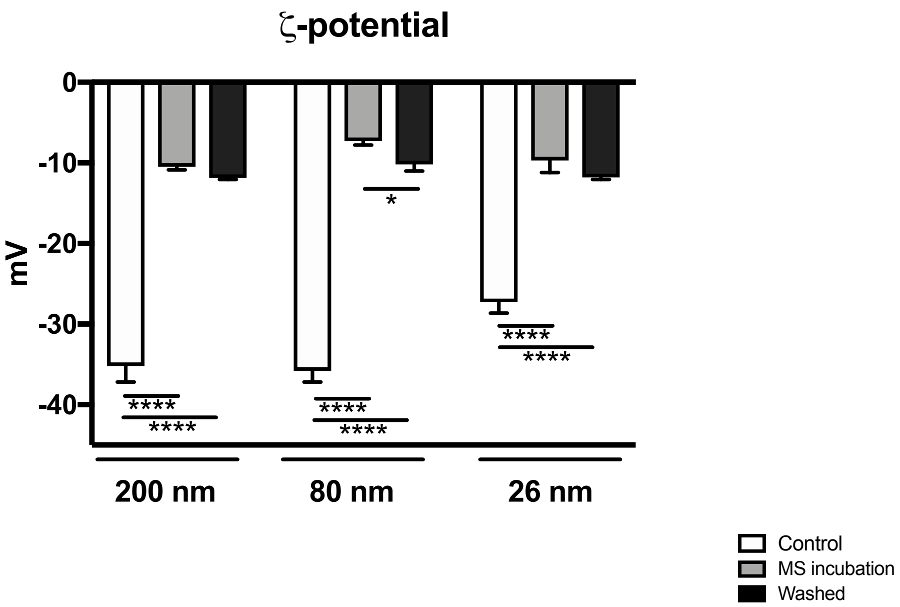

.

Supplementary Figure 5: Zeta-potential of 200, 80 and 26 nm COOH-PS before incubation (control), after 1h incubation with MS, and after 1h incubation and PBS washing. Incubations with MS were performed at 37°C. Each condition was set in triplicate. Bars correspond to means plus SD. Two-way ANOVA with Bonferroni post Hoc test. (*P<0,05; **** P<0,0001).

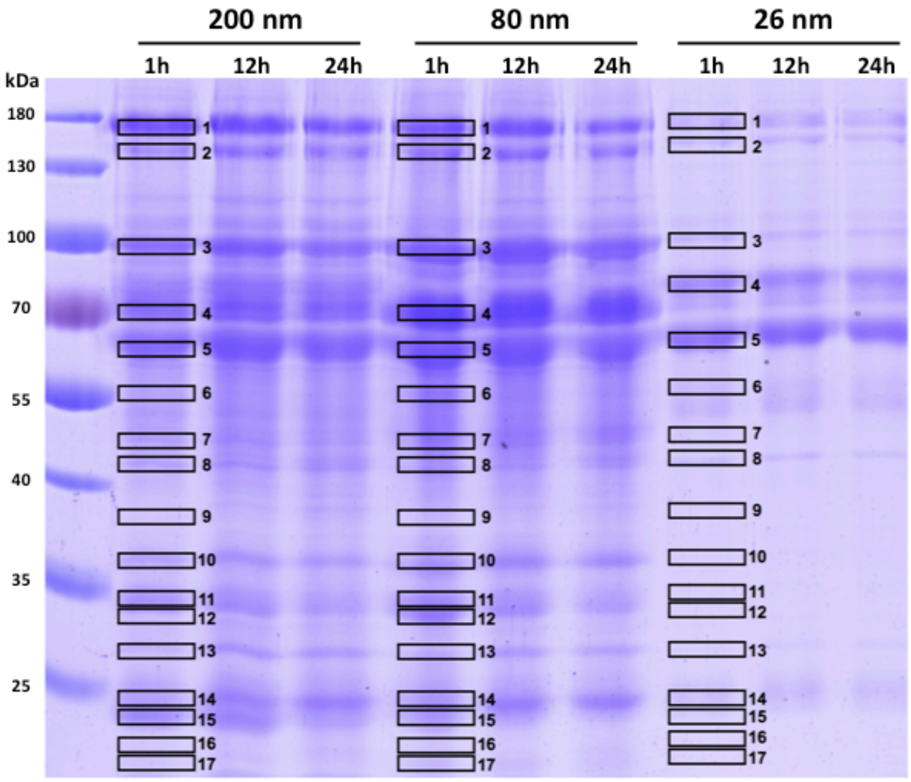

Supplementary Figure 6: Coomassie blue-stained SDS-gel showing the protein profiles of the corona formed when 0,5 mg ml^{-1} COOH-NPs (200 nm, 80 nm, 26 nm) were mixed with 50% mouse serum. The rectangles indicate the 17 gel slices which were individually analyzed by mass spectrometry.

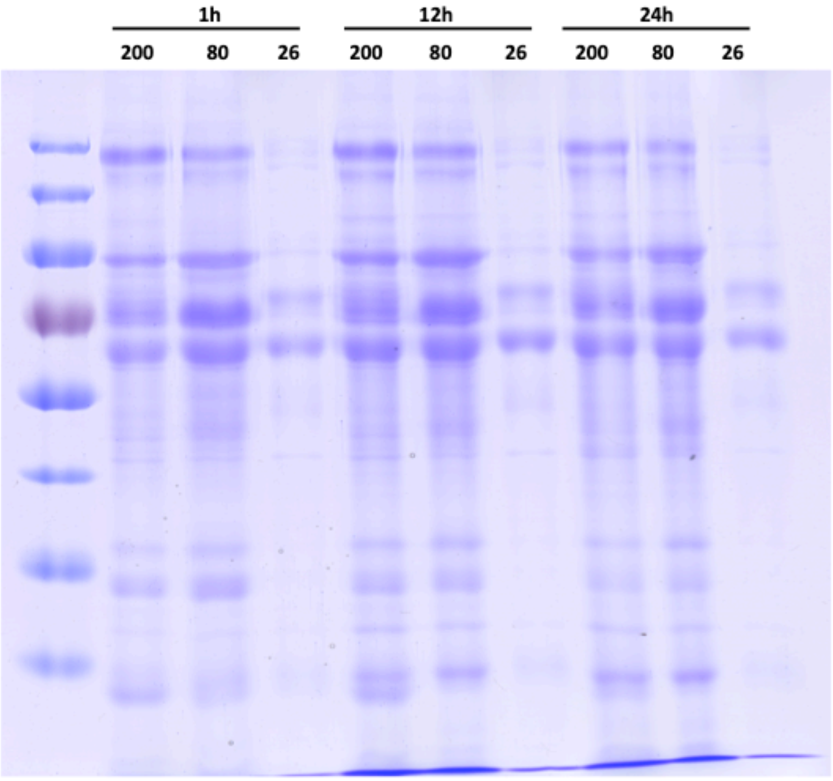

Supplementary Figure 7: Coomassie blue-stained SDS-gel showing the protein profiles of the corona formed when 0,5 mg ml^{-1} COOH-NPs (200 nm, 80 nm, 26 nm) were mixed with 50% mouse serum. NP were separated from free proteins by centrifugation and proteins adsorbed to COOH-PS NP was resolved by 10% SDS-Page gel and stained with Coomassie.

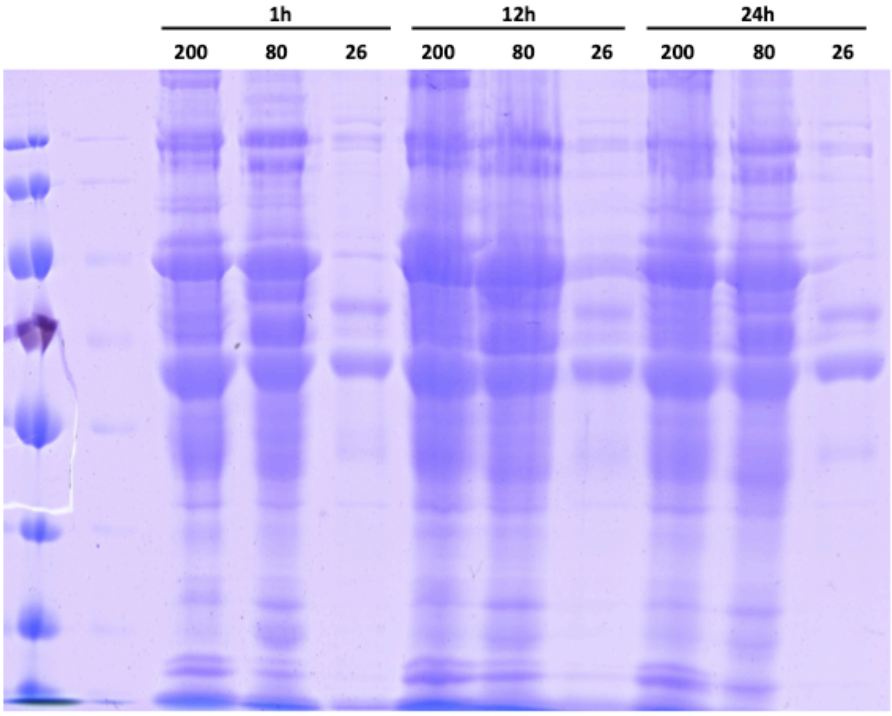

Supplementary Figure 8: Coomassie blue-stained SDS-gel showing the protein profiles of the corona formed when $5x10^{10}$ µm^2 COOH-NPs (200 nm, 80 nm, 26 nm) were mixed with 50% mouse serum. NP were separated from free proteins by centrifugation and proteins adsorbed to COOH-PS NP was resolved by 10% SDS-Page gel and stained with Coomassie.

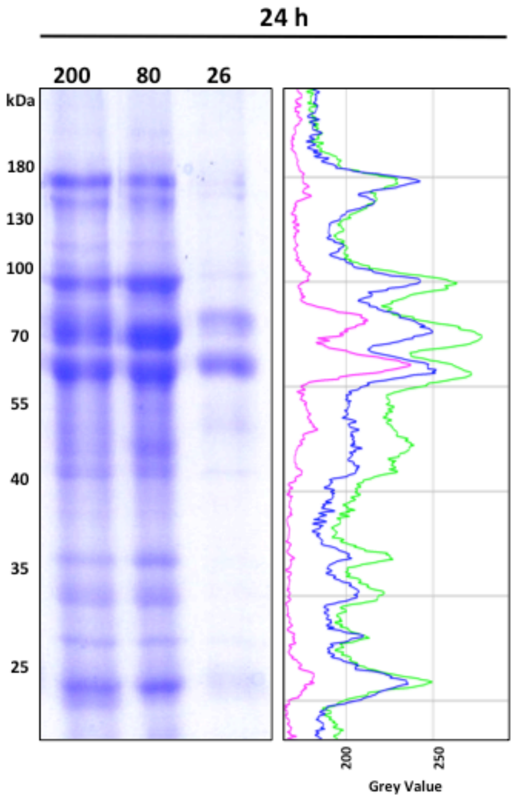

Supplementary Figure 9: Size-dependent differences around COOH-PS NP by SDS-PAGE gel. 200 nm, 80 nm and 26 nm COOH-PS NPs were incubated with MS at 0,5 mg ml^{-1} for 24h at 37ºC. The protein corona was resolved by 10 % acrylamide SDS-PAGE gel and a densitometry analysis using Image J /FIJI.

Supplementary Table 1: The top 20 most abundant serum proteins bound on the COOH-PS NP.

Selection of identified corona proteins after 1h incubation and washing. Murine serum proteins detected in the protein corona of COOH-PS NP determined by proteome analysis (mass spectrometry)

	200 nm COOH-PS		80 nm COOH-PS		26 nm COOH-PS	
		MW (kDa)		MW (kDa)		MW (kDa)
**1**	Myosin-9	226	Myosin-9	226	Murinoglobulin-1	165
**2**	Murinoglobulin-1	165	Talin-1	269	Apolipoprotein A-I	31
**3**	Serine protease inhibitor A3K	47	Apolipoprotein E	36	Alpha-2-macroglubulin	164
**4**	Talin-1	269	Serine protease inhibitor A3K	47	Gelsolin	85
**5**	Apolipoprotein E	36	Coagulation factor V	247	Apolipoprotein E	36
**6**	Antitrombin-III	52	Complement C3	186	Serotransferrin	77
**7**	Alpha-2-macroglubolin	164	Murinoglobulin-1	165	Serum albumin	69
**8**	Inter alpha-trypsin inhibitor, heavy chain 4	104	Antitrombin-III	52	Heat shock cognate	71
**9**	Serum albumin	69	Serum albumin	69	Transferrin receptor protein 1	85
**10**	Complement C3	186	Apolipoprotein B-100	509	14-3-3 protein zeta/delta	28
**11**	Apolipoprotein B-100	509	Alpha-2-macroglobulin	164	Ig mu chain C	50
**12**	Coagulation factor V	247	Inter alpha-trypsin inhibitor, heavy chain 4	104	Serine protease inhibitor A3K	47
**13**	Apolipoprotein A-I	31	Transferrin receptor protein 1	85	Integrin alpha-2	129
**14**	Carboxypeptidase N catalytic chain	51	Serotransferrin	77	Complement C3	186
**15**	Ig mu chain C region	50	Apolipoprotein A-I	31	Antitrombin-III	52
**16**	Heat shock cognate	71	Thrombospondin-1	129	Hemopexin	51
**17**	Serotransferrin	77	Gelsolin	85	Alpha-actin-1	103
**18**	Serine protease inhibitor A3M	47	EH domain-containing protein 4	61	Clusterin	52
**19**	Actin, cytoplasmic 1	42	Carboxypeptidase N catalytic chain	51	Complement C1q subcomponent subunit B	26
**20**	Ig kappa chain C region	12	Actin, cytoplasmic 1	42	Actin, cytoplasmic 1	42

Supplementary Box 1: Biological relevance of protein corona bound on the COOH-PS NP

Serum amyloid A-4 (SAA-4) and SAA-2 bound to only 80nm COOH-PS NP. SAA, a family of apolipoproteins associated with HDL were initially considered to play a key role in the pathogenesis of amyloid A-type amyloidosis, a complication of a number of inflammatory diseases and infections.[1] Moreover, APO A-II, APO C-I and also phosphatidylcholine-sterol acyltransferase (LCAT) bound uniquely to 80 nm COOH-PS NP. In fact, lipid metabolism-related proteins were marked present in MS corona of COOH-PS NP. Several apolipoproteins were identified in all sizes of NPs, but the relative APO E and APO A-I as compared with other apolipoproteins were much greater on the NP surface, suggesting a preferential binding of APO A-I and APO E. Apolipoproteins are known to exhibit a great binding affinity to NP surface compared with serum albumin, however, these proteins are less abundant and have slower binding kinetics which as observed in SDS-gels (Figure 3).[2] with the Apolipoproteins are involved in the transportation of lipids and cholesterol in the bloodstream, and are expected to greatly affect the intracellular tracking, fate, and transport of NP in cells and organs.[3] In particular, by binding with high affinity to cell-surface receptors, APO-E, a constituent of intermediate density lipoproteins (IDLs) is a key regulator of plasma lipid levels and actively contribute to homeostatic control of plasma and tissue lipid content.[4] APO-E exhibits isoform-specific effects on brain-blood barrier dysfunction and vascular dementia. [4, 5] Additionally, APO-E has been related to influence host susceptibility to parasitic (malaria)[6] bacterial (*Listeria monocytes*[7], *Klebsiella pneumonia*[8]) and viral infections (HSV[9], HIV[10, 11]). APO A-I is a major component of high-density lipoproteins (HDLs) and also forms part of chylomicrons and is responsible for solubilization of lipids in HDL particles. Modulatory effect on regulatory T cells, bactericidal and bacteriostatic ability and also anti-apoptotic functions was also associated with APO A-I.[12-14] Recently was studied as a therapeutic agent for cardiovascular disease and also described as an anti-tumorigenic agent.[10, 13-16]

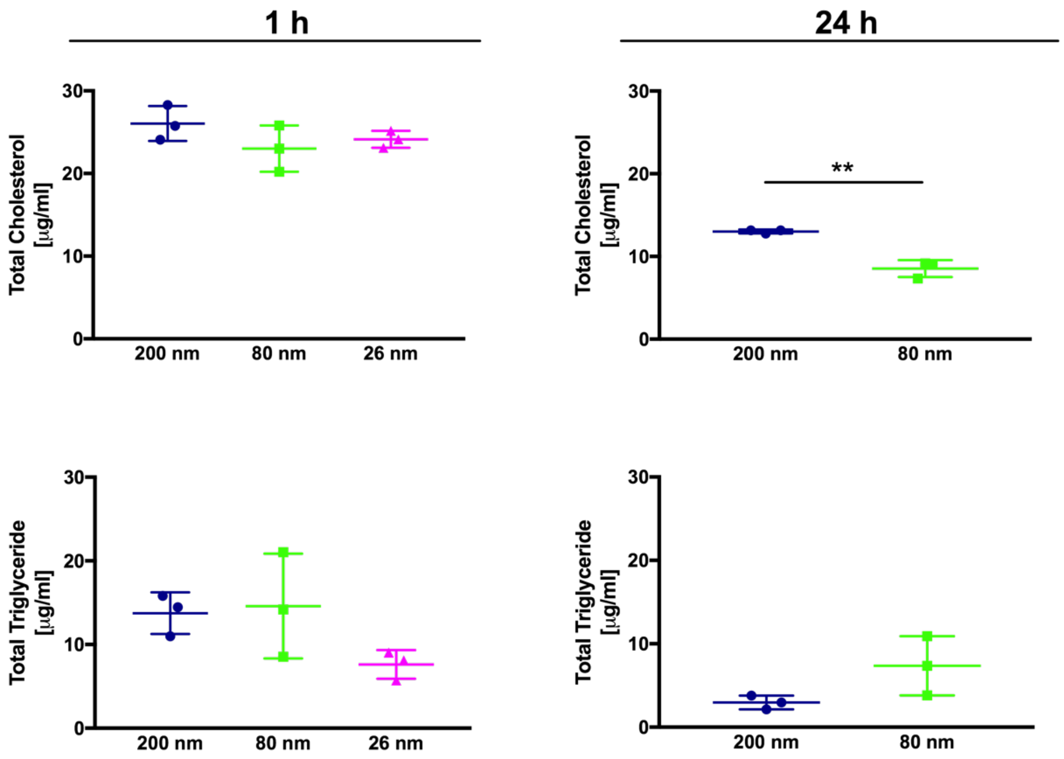

Supplementary Figure 10: Lipid quantification on COOH-PS NP after MS incubation. Corona levels of triglycerides and cholesterol were quantified after 1h and 24h incubation with MS in COOH-PS NP normalized to 0,5 mg ml^{-1} total mass. Each condition was set in triplicate. Bars correspond to means plus SD. One-way ANOVA with Bonferroni post Hoc test. (*P<0,05; **P<0,01; **** P<0,0001).

References

1. Bruun, C.F., K. Sletten, and G. Marhaug, *Mouse serum amyloid A (SAA) proteins isolated by two-dimensional electrophoresis: characterization of isotypes and the effect of separate and combined administrations of cytokines, dexamethasone and lipopolysaccharide (LPS) on serum levels and isotype distribution.* Clinical & Experimental Immunology, 1998. **111**(1): p. 231-236.
2. Schaffler, M., et al., *Serum protein identification and quantification of the corona of 5, 15 and 80 nm gold nanoparticles.* Nanotechnology, 2013. **24**(26): p. 265103.
3. Mortensen, N.P., et al., *Dynamic development of the protein corona on silica nanoparticles: composition and role in toxicity.* Nanoscale, 2013. **5**(14): p. 6372-80.
4. Mahley, R.W., K.H. Weisgraber, and Y. Huang, *Apolipoprotein E: structure determines function, from atherosclerosis to Alzheimer's disease to AIDS.* J Lipid Res, 2009. **50 Suppl**: p. S183-8.
5. Yamada, M. and H. Naiki, *Cerebral amyloid angiopathy.* Prog Mol Biol Transl Sci, 2012. **107**: p. 41-78.
6. Sinnis, P., *The malaria sporozoite's journey into the liver.* Infect Agents Dis, 1996. **5**(3): p. 182-9.
7. Roselaar, S.E. and A. Daugherty, *Apolipoprotein E-deficient mice have impaired innate immune responses to Listeria monocytogenes in vivo.* J Lipid Res, 1998. **39**(9): p. 1740-3.
8. de Bont, D.B., et al., *Increased stability of peptidesulfonamide peptidomimetics towards protease catalyzed degradation.* Bioorg Med Chem, 1999. **7**(6): p. 1043-7.
9. Itzhaki, R.F., et al., *Herpes simplex virus type 1 in brain and risk of Alzheimer's disease.* Lancet, 1997. **349**(9047): p. 241-4.
10. Gunnarsson, S.B., et al., *Analysis of nanoparticle biomolecule complexes.* Nanoscale, 2018. **10**(9): p. 4246-4257.
11. Burt, T.D., et al., *Apolipoprotein (apo) E4 enhances HIV-1 cell entry in vitro, and the APOE epsilon4/epsilon4 genotype accelerates HIV disease progression.* Proc Natl Acad Sci U S A, 2008. **105**(25): p. 8718-23.
12. Tada, N., et al., *Antimicrobial activity of lipoprotein particles containing apolipoprotein Al.* Mol Cell Biochem, 1993. **119**(1-2): p. 171-8.
13. Zamanian-Daryoush, M., et al., *The cardioprotective protein apolipoprotein A1 promotes potent anti-tumorigenic effects.* J Biol Chem, 2013. **288**(29): p. 21237-52.
14. Lara, S., et al., *Identification of Receptor Binding to the Biomolecular Corona of Nanoparticles.* ACS Nano, 2017. **11**(2): p. 1884-1893.
15. Camont, L., M.J. Chapman, and A. Kontush, *Biological activities of HDL subpopulations and their relevance to cardiovascular disease.* Trends Mol Med, 2011. **17**(10): p. 594-603.
16. Nam, N.N. and S.Y. Han, *Formation of High-Density Lipoprotein (HDL) Coronas on SilicaNanoparticles Occurs by Adsorption of Intact HDL Particulate.* Bulletin of the Korean Chemical Society, 2015. **37**: p. 3-4.
